# Cross-cultural perspectives on the patient-provider relationship: a qualitative study exploring reflections from Ghanaian medical students following a clinical rotation in the United States

**DOI:** 10.1186/s12909-015-0444-9

**Published:** 2015-09-28

**Authors:** Nauzley C. Abedini, Sandra Danso-Bamfo, Joseph C. Kolars, Kwabena A. Danso, Peter Donkor, Timothy R. B. Johnson, Cheryl A. Moyer

**Affiliations:** 1 University of Washington Internal Medicine Residency Program, Seattle, WA USA; 2 Harvard T.H. Chan School of Public Health, Boston, MA USA; 3 Department of Internal Medicine and Global REACH, University of Michigan Medical School, Ann Arbor, MI USA; 4 School of Medical Sciences, College of Health Sciences, Kwame Nkrumah University of Science and Technology, Kumasi, Ghana; 5 Department of Obstetrics and Gynecology, University of Michigan Medical School, Ann Arbor, MI USA; 6 Departments of Learning Health Sciences and Obstetrics and Gynaecology, University of Michigan Medical School, Ann Arbor, MI USA

**Keywords:** International partnerships, Patient-provider relationship, Patient-provider communication, Global health, Humanism in medicine, Clinical electives, Medical education

## Abstract

**Background:**

In international health experiences, learners are exposed to different culturally-based patient care models. Little is known about student perceptions of patient-provider interactions when they travel from low-to high-resource settings. The purpose of this study was to explore these reflections among a subset of Ghanaian medical students who participated in clinical rotations at the University of Michigan Medical School (UMMS).

**Methods:**

In-depth, semi-structured interviews lasting 60–90 min were conducted with 15 individuals who had participated in 3-to 4-week clinical rotations at UMMS between January 2008 and December 2011. Interviews were conducted from March to August 2012 and transcribed verbatim, then independently coded by three investigators. Investigators compared open codes and reached a consensus regarding major themes.

**Results:**

Participating Ghanaian medical students reported that their perspectives of the patient-provider relationship were significantly affected by participation in a UMMS rotation. Major thematic areas included: (1) observations of patient care during the UMMS rotation, including patient comfort and privacy, physician behavior toward patients, and patient behavior; (2) reflections on the role of humanism and respect within patient care; (3) barriers to respectful care; and (4) transformation of student behaviors and attitudes. Students also reported integrating more patient-centered care into their own medical practice upon return to Ghana

**Discussion:**

Participation in a US-based clinical rotation has the potential to introduce medical students from resource-limited settings to a different paradigm of patient-provider interactions, which may impact their future behavior and perspectives regarding patient care in their home countries.

**Conclusions:**

Students from under-resourced settings can derive tremendous value from participation in clinical electives in more affluent settings, namely through exposure to a different type of medical care.

## Background

International health electives have become increasingly popular amongst medical students and have a number of documented benefits for participating students, including an increased likelihood of choosing a career in primary care or public service [1, 2], improved physical examination and history-taking skills, a greater appreciation of public health and the importance of socioeconomic influences on health [2, 3], exposure to different perspectives and health care systems, and personal growth [3]. However, the vast majority of international electives involve students from resource-rich settings traveling to resource-poor settings [2, 4, 5]. As such, studies seeking to ascertain the value and impact of such experiences have generally not captured the perspectives of medical trainees from under-resourced settings who partake in electives in more affluent regions [6].

Furthermore, the encounters from which many of these learning outcomes are derived generally involve learner observation of the interactions between patients and providers in foreign contexts. The nature of this relationship is often affected by the culture of both the patient and provider [7–9]. In the US, shared-decision making and egalitarian approaches have been adopted based on societal norms [10], while in other parts of the world, more paternalistic models are accepted due to cultural standards. Little is known about how observations of the former could impact a medical trainee from the latter context.

Since 2007, the University of Michigan Medical School (UMMS) has sponsored a bilateral exchange program between UMMS students and medical students from universities in Ghana. A quantitative survey of Ghanaian medical students who previously participated in 3-to 4-week clinical rotations at UMMS demonstrated that students came away with improved knowledge of US medical care, made changes to their approaches to patient care, and felt better equipped to serve the people in their home community as a result of the experience [11]. However, the specific experiences and exposures that led to behavioral and attitudinal shifts regarding patient care were not elucidated. As such, we sought to use qualitative interviews with a subset of Ghanaian medical students who had formerly participated in a UMMS-based clinical elective to perform an in-depth exploration of their perceptions regarding patient care and the patient-provider relationship.

## Methods

### Setting

A pathway has been available since 2007 for Ghanaian medical students to participate in a 3-to 4-week clinical elective a UMMS, usually during their second clinical year (fifth year of a 6-year training program). A quantitative online survey of 73 Ghanaian medical students who had participated in a UMMS-based elective demonstrated that students came away with different behaviors and attitudes regarding patient care [11], the details of which were not captured using the numerical coding system. Thus, we sought to explore the themes of patient care and patient-provider relationship using qualitative, in-depth interviews.

### Participants

Between January 2008 and December 2011, 102 Ghanaian medical students participated in a clinical elective at UMMS. Seventy-three of these individuals had previously participated in the online quantitative survey exploring their perceptions of the impact of their rotation [11]. Given the desire to further explore the question of how the UMMS elective impacted their perceptions of patient care and the patient-provider relationship, all 102 individuals were invited to participate in in-depth interviews. These individuals had similar demographic and educational backgrounds and took part in similar clinical experiences at UMMS, ensuring homogeneity of the sampling pool.

Participation in the interview was voluntary. Individuals who either declined the offer to participate or who did not respond after three separate contact attempts (emails sent approximately 2 to 4 weeks apart) were excluded from the study. All study participants gave their consent electronically and verbally prior to participation in the interview.

This study was reviewed and performed under an exemption granted by both the Ethical and Protocol Review Committee of the University of Ghana Medical School as well as the University of Michigan Health Sciences and Behavioral Sciences Institutional Review Board.

### Data collection

Interviews and coding were performed in an iterative fashion consistent with qualitative methodology. As such, no pre-determined sample size was set. Interviews were performed with a random subset of individuals who agreed to participate. The study cohort was somewhat limited due to scheduling difficulties related to geographic discordance with the interviewer and/or time limitations while on busy clinical services), and as such, not all individuals who agreed to participate could actually be interviewed.

A qualitative approach was utilized because of its ability to identify and explore in-depth the meaning that individuals confer on their subjective experience [12]. Between March and August 2012, one investigator (SDB) conducted individual, face-to-face, semi-structured interviews lasting 60- to 90-min with 15 individuals who had participated in 3-to 4-week clinical rotations at UMMS between January 2008 and December 2011. Participants were offered a complimentary meal (approximately $8-10 USD). Interviews were conducted and concomitant coding was performed until saturation of themes was achieved, thus determining the sample size of 15 participants.

Each interview was based on a 35-question interview guide designed specifically for this study. The major questions from the interview guide that were relevant to this study are summarized in Table 1. The questions were deliberately open-ended and interviews were semi-structured, allowing the interviewer the flexibility to probe into specific themes. The interview guide was developed based on findings from a previous review of the literature surrounding the impact of international experiences conducted by one of the authors (CAM) [3], exploratory interviews with one investigator who had previously participated in the exchange program (SDB), and informal discussions with current exchange participants at UMMS. This was followed by an iterative process of expert review amongst two investigators (KAD, TRBJ) and a third party who had been directly involved in the formation and execution of the exchange at all three institutions, and who had engaged with participating students on a personal and/or professional level. Drafted questions were ultimately selected for inclusion based on a consensus among all investigators. The final tool was designed to be flexible to allow the investigator to probe participant responses.

**Table 1:** Summary of interview questions used during semi-structured interviews with the 15 study participants

1.	Prior to your rotation at the University of Michigan, did you participate in any international electives or exchanges?
	a. If so, where?
	b. Describe your experience.
	c. Did this effect your decision to pursue the University of Michigan rotation? If so, how?
2.	After participation in the University of Michigan rotation did you participate in any international electives or exchanges?
	a. If so, where?
	b. Describe your experience.
	c. Was your decision to participate in additional international experiences in anyway prompted by your experience at the University of Michigan? If so, how?
3.	What were your first impressions when you began you rotation, particularly with regards to clinical exposure?
4.	Did any part of your experience in Michigan surprise you? Why were you surprised?
5.	Did anything surprise you about yourself?
6.	Did you feel adequately prepared for what you encountered during your time in Michigan?
7.	What did you learn from your experience in Michigan?
8.	What aspect(s) of medical care at the University of Michigan is/are different from other experiences you have had?
9.	Did you learn anything at the University of Michigan that you can apply to your current medical training/practice?
10.	How did participation in the Michigan exchange affect you personally?
11.	Has the Michigan exchange affected your approach to patient care? If so, how?
12.	What impact, if any, has your Michigan rotation experience had on your post-graduation plans?
13.	In retrospect, what was the most valuable thing that you took away from your experience?

**Table 2:** Demographics of the 15 individuals who participated in in-depth interviews

Gender	Male: 47 % (7/15)
	Female: 53 % (8/15)
Average age of study participants	25.9 ± 1.4 years
	(Range: 24–29 years)
Study participants’ home institution at time of participation in UMMS exchange	KNUSTSMS: 40 % (6/15)
	UGMS: 33 % (5/15)
	UDSSMS: 27 % (4/15)
Average time from rotation end-date to study participation	17.3 ± 9.5 months
	(Range: 8–40 months)
Departments at UMMS where study participants rotated^a^	Obstetrics and Gynecology: 50 % (9/18)
	Pediatrics: 17 % (3/18)
	Other^b^: 37 % (6/18)
Did the student participate in a previous international exchange prior to UMMS rotation?	No: 100 % (15/15)

^a^Three students participated in rotations in multiple departments. One student went on two separate rotations to UMMS

^b^Includes Departments of Pediatric Infectious Disease (1), Anesthesiology (1), Internal Medicine (1), Radiology (1), Family Medicine (1), Physical Medicine and Rehabilitation, and Cardiology/Electrophysiology (1)

### Data analysis

Interviews were audio-recorded in English and transcribed verbatim into Microsoft Word. Transcripts were reviewed for completeness and accuracy by the interviewer (SDB) and all identifiers were removed prior to distribution to the co-investigators to maintain participant anonymity. Transcripts were then read and independently coded by three investigators (NCA, SDB, and CAM), focusing initially on the identification of broad thematic areas consistent with an inductive approach. Based on findings of initial coding, interview questions were adjusted to better develop and elaborate on emerging themes. Interviews were continued until saturation of themes was achieved. The investigators discussed the themes identified and created a preliminary codebook based on consensus that was used to guide subsequent coding.

The results presented here focus on all data pursuant to the broad category of “patient-provider relationship.” One of the investigators (NCA) re-coded electronic copies of the transcripts in NVivo 9 (QSR International, Victoria, Australia) using focused, line-by-line analysis based on the “patient-provider relationship” codes identified in the initial round of coding. Two of the investigators (NCA, CAM) met weekly to discuss this second round of coding, resolve any uncertainties surrounding codes assigned, and ensure that coding was true to the codebook. Additional coding categories were identified, discussed amongst the investigators, and added to the codebook as necessary.

## Results

Of the 102 students who participated in a UMMS rotation from January 2008 to December 2011, 33 (32 %) agreed to participate in one-on-one interviews. Fifteen interviews were subsequently performed with consented individuals based on ability of participants who were geographically dispersed around Ghana to connect with the interviewer. Saturation of themes was achieved after 15 interviews. On average, study participants were interviewed 17.3 months after completing their UMMS clinical elective. Participant demographics are summarized in Table 2. All participants came from one of the three public medical schools in Ghana: Kwame Nkrumah University of Science and Technology School of Medical Sciences (KNUSTSMS), University for Development Studies School of Medical Sciences (UDSSMS) or the University of Ghana Medical School (UGMS).

All participating Ghanaian medical students reported that their perspectives of the patient-provider relationship were significantly affected by participation in a UMMS rotation. Systematic coding yielded the following themes: (1) observations of patient care during the UMMS rotation, which included observations about patient comfort and privacy, physician behavior toward patients, and patient behavior; (2) reflections on the role of humanism and respect within patient care; (3) barriers to respectful care; and (4) transformation of student behaviors and attitudes.

### Observations of patient care during the UMMS rotation

Students observed several differences between patient care in the US compared to practice in Ghana. First, the majority of students observed different values and behaviors regarding patient comfort and privacy: “I learned that there is another aspect to medicine aside [from] just giving your medication or doing your surgeries. Psychologically, if you made the person comfortable then you’d also be helping and treating the patient” (26-year-old female from KNUSTSMS).

Additionally, students observed differences in physician behavior toward patients, specifically with regard to shared-decision making and protecting patient autonomy.

“I realized in Michigan that the patient comes like a customer, and then the doctor is the one who is sort of selling to the patient. So you try as much as possible to be very polite, very nice, listen to the patient, give options, let the patient make choices—which I think is very good medicine to practice. But in Ghana … the patient entrusts their life with you. Therefore you are God, you decide whatever—everything—for them” (29-year-old male from UGMS).

Students also perceived differences in patients’ attitudes around illness, which they believed influenced the patients’ ability and likelihood to participate in medical decision-making. For example, students noted that patients were more apt to ask questions of the provider in the US, which was attributed to higher education amongst US patients. One student shared observations of parents during a pediatric clinic visit at Michigan: “[The parents] would research on the [inter]net … and they already have a list of questions … and that I think was quite impressive and that’s one thing that I think we should encourage for the parents [in Ghana]” (27-year-old female from UDSSMS). Another student spoke of the different patient attitudes around illness, drawing comparisons based on her observations of parent views toward having a child with a congenital anomaly:

“[At Michigan] the parents show so much love and concern and it really touched my heart. … I mean here [in Ghana], because of our belief system and stuff, you have a child like that, the first thing they might say is, ‘It’s a taboo,’ or ‘The gods are angry,’ or something, so you tend to neglect the child” (27-year-old female from UDSSMS).

Thus, cultural taboos and associated feelings of disempowerment were seen as barriers to patient participation in medical care in Ghana, while education and access to information in the US allowed patients to engage more actively in the management of their disease.

### Reflections on humanism and respect in patient care

The majority of study participants spoke of increased appreciation for the role of respect and empathy within medical care:

“[W]hat I saw from the interaction between workers, health workers and patients [at UMHS] was a certain level of respect. You wouldn’t see a doctor or a nurse being rude to a patient or shouting at him. I really hope to put this into practice because, a lot of times, our patients [in Ghana] just come and they need someone to be nice to them. … I hope to be a very good doctor, putting into practice a few of things I observed at Michigan” (25-year-old male from KNUSTSMS).

In fact, for some students, these observations sparked reflections on ethical dilemmas they encountered at their home institutions:

“…[In Ghana] we don’t have that respect. People just trample on patients’ rights. Sometimes the patient is genuinely demanding something that is due her, but because of our system, because of the lack of respect you have, the person will not get it…. I don’t think our system is right” (25-year-old female from KNUSTSMS).

Another student remarked, “I think that patients deserve more than we give them here [in Ghana]. You have to respect them” (25-year-old female from KNUSTSMS).

### Barriers to respectful care

While recognizing the importance of humanism and respect within medical practice, the majority of students identified a variety of environmental barriers to practicing respectful care in Ghana:

“…[S]ometimes it is very hard because you have a lot of patients, because of our literacy level here [in Ghana] as opposed to that outside. It’s very hard to explain exactly what you are going to do for a patient and what is wrong with them for them to understand it…. Mainly what they are interested in is getting better. Once they are better, then fine, that’s it. What is wrong, their options—yes, that is hard here” (26-year-old female from KNUSTSMS).

In addition to high caseloads and low literacy levels amongst patients in Ghana, students also cited additional barriers, including resource constraints and personal discomfort arising from discussing emotionally charged topics with patients.

### Transformation of student behaviors and attitudes

Nearly all students translated some of the observations they made at UMMS regarding the patient-provider relationship into attitudinal and behavior changes upon return to their home institution. First, students determined that employing more humanistic, respectful care was feasible despite environmental barriers: “…[W]e don’t have technology, we don’t have infrastructure, but in terms of our relationship with our patients, we don’t have to have fancy gadgets before we can make our patients feel comfortable in the setting that we have here” (26-year-old female from KNUSTSMS).

In an effort to enhance their relationship with patients, many students stated that they had made changes to their patient communication style as a result of their UMMS rotation: “What I learned from [Michigan] was to … simplify what [the patient’s] condition is so that they will be aware of what they have in terms of taking care of themselves or preventing any complications” (24-year-old female from KNUSTSMS). Others referred to greater inclusion of patients within their care by providing opportunities for questions or shared decision-making: “I actually ask them if they have any questions when I’m done taking my histories and I actually take time to answer the ones that I know” (27-year-old female from KNUSTSMS). These changes were grounded in a greater sense of empathy and connection to patients. One student summarized the evolution in her approach to patient care as “blending your … professional work with love…. Because sometimes I think there is a tendency to see your patients as your job…. [E]ven though you’re supposed to emotionally detach yourself, you still do your work out of love for them” (27-year-old female from UDSSMS).

## Discussion

This study provided a unique glimpse into the experiences and attitudes of Ghanaian students regarding patient care and the patient-provider relationship after participation in a US-based clinical elective. They observed a different paradigm of patient care and cited a number of differences in the patient-provider relationship compared to approaches they had encountered in their home country. Exposure to different frameworks for health care practice has previously been cited as a benefit of participation in international health electives [1–3], but generally from the perspective of medical trainees traveling from the global North to the global South. Here we demonstrate that reciprocal elective opportunities can have benefits for trainees coming from under-resourced settings to more affluent care settings–namely, by prompting reflection about differences in health systems, patient care and trainees’ professional duties as providers. To our knowledge, this is the first study to document observations of the culturally-based differences in the patient-provider relationship from the perspective of trainees from resource-poor settings.

Studies in the US looking at the effects of culture, race/ethnicity, and gender on patient satisfaction and outcomes have suggested that cultural congruence between providers and patients is a key to positive interactions [7–9]. In this study, however, the differences observed in the patient-provider relationship prompted ethical reflections for many study participants regarding the role of respect and humanism in patient care. Many study participants alluded to having observed interactions at their home institutions between patients and providers that, in their mind, constituted disrespectful care, even if they were condoned as normative practices based on societal or cultural values. Often this posed an ethical conundrum for students seeking to uphold high standards of humanism and professionalism. Such observations of lapses in professionalism and encroachment on patient rights have previously been documented in Africa [13, 14] as well as in Western settings [15–18]. Role-modeling and structured reflection have been identified as means of engendering professional values of empathy, altruism, and shared-decision making in a variety of contexts [19–21]. Future efforts should continue to aim to develop and implement formal curricula around these issues, while trainees are both abroad and at their home institutions.

Students identified a variety of environmental barriers to practicing respectful care in Ghana, including high caseloads, resource constraints, low patient education, and personal discomfort. Previous studies have identified these same environmental barriers to medical trainee professionalism education and practicing respectful care in under-resourced settings [19, 22, 23]. Other barriers include cultural traditions of communalism, which has been known to lead to compromise of patient confidentiality [19, 24], as well as established hierarchy and paternalism within medicine, which may impede patients from inquiring about their health conditions and thus compromise health literacy [19, 25]. However, despite the barriers that were identified, the ethical queries that arose from observing different approaches to patient care became the basis for lasting attitudinal and behavioral changes. In fact, improving patient-provider interactions in Ghana was cited as one of the most salient learning outcomes of participation in a US-based elective. These reflections and resulting shifts in attitudes and behaviors suggest that immersion activities in different contexts may have tremendous transformative capacity [26–28]. For many, these ethical dilemmas sparked an internal paradigm shift, in which students felt they could make changes to their own behaviors and attitudes, and also feasibly make changes on an institutional or even system level, to institute more respectful care. These changes had continued an average of 17 months after students had returned to their home institution. There is a need for further longitudinal studies to see if these attitude/behavioral shifts have greater longevity. Furthermore, while confrontation with new environments and contexts can be one means of invoking changes in attitude, this does not suggest that it is the only way to trigger ethical inquiry. Rather, further efforts should be made to compare formal and informal curricula around professionalism and patient-provider communication in a variety of contexts and thus identify and nurture those components of medical training that promote humanistic medical practice.

### Study limitations

One limitation of the study is the small sample size, though this was largely mitigated by the use of qualitative methodologies and the fact that 15 interviews were required to achieve saturation of themes. An additional limitation was that selection criteria for participation was based on a convenience sampling as a number of students who had participated in the UMMS elective had since graduated and were working in decentralized locations around Ghana. Furthermore, many were house officers at time of interview and thus had rigid schedules that inhibited their ability to meet with our interviewer. However, all participants had no prior clinical elective experience outside of Ghana aside from their UMMS rotation and also had fairly standardized clinical exposures while at UMMS, and thus the reflections offered were based on generally uniform exposures at a single institution. Another potential limitation of this study is that results were gathered via face-to-face interviews, and respondents may have skewed their responses in the name of social desirability. However, these limitations were mitigated by the fact that all data were collected by a Ghanaian interviewer who was a medical student at the time and thus junior to those being interviewed. She was trained in qualitative interviewing techniques to minimize the potential for interviewer-introduced bias. We believe these efforts minimized the risk of social desirability bias.

## Conclusions

Our study demonstrates that students from under-resourced settings can derive tremendous value from participation in clinical electives in more affluent settings, which is largely predicated on exposure to a different type of medical care. Such experiences have the capacity to change attitudes and behaviors regarding the patient-provider relationship, changes that persisted nearly a year-and-a-half after the initial experience abroad. Future research should be directed at how such attitudinal shifts can be sustained long-term and also to develop locally based curricula that nurture humanistic medical practice amongst trainees.

## Abbreviations

UMMS: University of Michigan Medical School
UGMS: University of Ghana Medical School
UDS-SMHS: University of Development Studies School of Medical and Health Sciences
KNUST-SMS: Kwame Nkrumah University of Science and Technology School of Medical Sciences
